# Antenatal Steroids and the IUGR Fetus: Are Exposure and Physiological Effects on the Lung and Cardiovascular System the Same as in Normally Grown Fetuses?

**DOI:** 10.1155/2012/839656

**Published:** 2012-11-22

**Authors:** Janna L. Morrison, Kimberley J. Botting, Poh Seng Soo, Erin V. McGillick, Jennifer Hiscock, Song Zhang, I. Caroline McMillen, Sandra Orgeig

**Affiliations:** ^1^Early Origins of Adult Health Research Group, School of Pharmacy and Medical Sciences, Sansom Institute for Health Research, University of South Australia, GPO Box 2471, Adelaide, SA 5001, Australia; ^2^Molecular and Evolutionary Physiology of the Lung Laboratory, School of Pharmacy and Medical Sciences, Sansom Institute for Health Research, University of South Australia, GPO Box 2471, Adelaide, SA 5001, Australia

## Abstract

Glucocorticoids are administered to pregnant women at risk of preterm labour to promote fetal lung surfactant maturation. Intrauterine growth restriction (IUGR) is associated with an increased risk of preterm labour. Hence, IUGR babies may be exposed to antenatal glucocorticoids. The ability of the placenta or blood brain barrier to remove glucocorticoids from the fetal compartment or the brain is compromised in the IUGR fetus, which may have implications for lung, brain, and heart development. There is conflicting evidence on the effect of exogenous glucocorticoids on surfactant protein expression in different animal models of IUGR. Furthermore, the IUGR fetus undergoes significant cardiovascular adaptations, including altered blood pressure regulation, which is in conflict with glucocorticoid-induced alterations in blood pressure and flow. Hence, antenatal glucocorticoid therapy in the IUGR fetus may compromise regulation of cardiovascular development. The role of cortisol in cardiomyocyte development is not clear with conflicting evidence in different species and models of IUGR. Further studies are required to study the effects of antenatal glucocorticoids on lung, brain, and heart development in the IUGR fetus. Of specific interest are the aetiology of IUGR and the resultant degree, duration, and severity of hypoxemia.

## 1. Use of Antenatal Glucocorticoids for Fetal Lung Maturation in Women at Risk of Preterm Delivery 

In Australia, 8% of the 250,000 annual births are preterm, defined as delivery prior to 37-week completed gestation [1]. Furthermore, 7% of babies are born with intrauterine growth restriction (IUGR) [2], defined as a birth weight <10th centile [3–5] with the incidence of IUGR increasing with increasing prematurity [6, 7]. Preterm infants represent 75% of all neonatal deaths in Australia, with the vast majority of these deaths being due to pulmonary disease [1]. The costs of caring for preterm infants are high, $5.8 billion in the USA, representing 57% of neonatal care costs in that country [8]. The cost to support a single infant born at 25 weeks of gestation is estimated at $US 250,000 [9]. 

Glucocorticoids are administered [10] to pregnant women at risk of preterm labour occurring after 24 weeks of gestation to promote surfactant production and maturation of the fetal lung in order to make a successful transition to air-breathing. Experimental studies show improvement in fetal lung mechanics [11], increases in surfactant lipids and proteins [12, 13], and concomitant alterations in lung structure [13]. Antenatal glucocorticoid administration to women at risk of preterm labour reduces the incidence of neonatal respiratory distress syndrome (RDS) by *∼*35–45% [14]. Furthermore, antenatal glucocorticoids have been shown to reduce overall neonatal mortality and the need for neonatal respiratory support in preterm infants [15–18]. The effectiveness of antenatal glucocorticoids for promoting lung maturation declines after 7 days, and thus in practice women who continue to be at risk of preterm delivery have in the past been treated with repeated weekly doses of antenatal glucocorticoid therapy [16]. Despite little evidence from either clinical trials or animal studies of improved neonatal outcomes for repeated as opposed to single doses of glucocorticoid therapy [19], the practice became widespread. For example, obstetricians in Australia in 1998 [20] and the UK in 1999 [21] reported that if the risk of preterm delivery persisted, 85 and 98%, respectively would prescribe multiple courses of glucocorticoids. In 2001, a National Institutes of Health Consensus Group questioned the use of multiple-dose glucocorticoid therapy [22, 23] based on animal and human studies which indicated adverse effects after repeated courses of glucocorticoids, including fetal growth restriction and poorer neurodevelopmental outcome [24–29]. The group consequently recommended that prescribing of multiple doses be restricted to patients enrolled in clinical trials specifically designed to determine the risk/benefit ratio of multiple as opposed to single doses of glucocorticoids [22, 23]. However, more recently, in 2004, an European study determined that 85% of obstetric units continued to prescribe multiple courses of antenatal glucocorticoids, despite the fact that the risk/benefit ratio of multiple versus single doses was not yet known [30]. 

Recently, the first results of the randomised controlled trials of repeat- versus single-dose glucocorticoid therapy have been released [31]. The latest Cochrane review on the use of repeat doses of prenatal corticosteroids concludes that the short-term benefits which include a further reduction of 17% in the incidence of respiratory distress and a 16% reduction in serious health problems in the first weeks of life support the use of repeat doses, despite a small associated reduction in size at birth [32]. Follow-up studies of babies to two years of age show no significant harm in early childhood, but also show no benefit [32]. These results, together with the already widespread use of repeat antenatal glucocorticoid therapy, suggest that the practice is likely to continue to increase. Hence, it would appear prudent to heed the earlier recommendation that studies in animals be performed to determine the pathophysiological and metabolic consequences of repeat antenatal glucocorticoid treatment [22, 23]. This may be especially important in a subset of preterm infants that may be particularly vulnerable to glucocorticoid therapy.

IUGR due to placental insufficiency occurs when substrate supply is reduced and does not meet fetal demands. Hence, causes of IUGR include fetal factors (e.g., chromosomal abnormalities), maternal factors (e.g., undernutrition), environmental factors (e.g., high altitude), placental factors (e.g., placental infarction), and other factors (e.g., reproductive technologies) [4, 5, 33, 34]. As IUGR is also associated with an increased risk of preterm labour [6, 35], IUGR babies may also be exposed to antenatal glucocorticoids [7]. Recently, we [33, 36] and others [37–42] have asked whether exposure of IUGR fetuses to antenatal glucocorticoids is beneficial in terms of their cardiovascular, neurological, and respiratory development. Early studies demonstrated that lung growth and surfactant production were each accelerated in IUGR fetuses in the absence of antenatal glucocorticoid treatment [43]. This was thought to be a result of the elevated plasma cortisol levels present in IUGR fetuses [44]. Other studies have also reported that there is no evidence that the incidence of RDS is lower in IUGR babies [13, 45, 46], and conversely others have shown that there is an increased risk of RDS in IUGR babies [47]. There are conflicting data on whether antenatal glucocorticoids are [37] or are not [48] associated with a reduction in the complications associated with preterm delivery in IUGR fetuses. A large study of 19,759 very-low-birth-weight neonates found that antenatal glucocorticoids lowered the risk of RDS, intraventricular haemorrhage, and perinatal death in both normally grown and growth restricted fetuses [37]. On the other hand, a study of 1148 neonates found that there was no difference in the incidence of RDS, intraventricular haemorrhage or necrotizing enterocolitis in growth-restricted fetuses whether they were treated with antenatal glucocorticoids or not [48]. The reported differences in the effectiveness of antenatal glucocorticoids on neonatal outcome in normally grown compared to IUGR babies may be due to differences in either the degree or duration of exposure to antenatal glucocorticoids or the effects of glucocorticoids (endogenous or exogenous) on the development of organ systems in the normally grown compared to the IUGR fetuses. In this paper we examine whether the physiological exposure to, and the effects of, antenatal glucocorticoids are the same in normally grown and IUGR fetuses, focusing on a range of animal studies used in our laboratory. Firstly, this paper will discuss the effects of reduced fetal growth on the expression of drug transporters that remove glucocorticoids from the fetal compartment and, thus, influence the degree and duration of fetal exposure to glucocorticoids. Secondly, we will review the effects of IUGR on lung and cardiovascular development and the impact of glucocorticoids on these key organ systems in the IUGR sheep fetus.

## 2. Exposure of the Fetus to Antenatal Glucocorticoids

Cortisol, the predominant form of active glucocorticoid in humans, guinea pigs, and sheep, interacts with the glucocorticoid receptor (GR) in target cells to ensure functional maturation of fetal organs such as lung, liver, gut, and kidney which are necessary for survival of the newborn [49–51]. For example, cortisol increases pulmonary surfactant synthesis and secretion as well as structural maturation of the alveoli to support postnatal lung function [51, 52]. Clinically, women are treated with either dexamethasone or betamethasone, both fluorinated corticosteroids that cross the placenta and have identical genomic effects [53]. However, as betamethasone is more potent than dexamethasone in terms of metabolic nongenomic effects, it is the drug of choice [53]. The synthetic corticosteroids are significantly more effective than hydrocortisone as they are not inactivated by endogenous dehydrogenase enzymes (see below) [54]. Hence, in humans a very high dose of hydrocortisone is required as an alternative to dexamethasone or betamethasone, and in sheep high doses of maternal hydrocortisone do not promote lung maturation [55]. While glucocorticoids are necessary for survival of the fetus, exposure to excess endogenous or exogenous glucocorticoid in a healthy fetus has also been associated with fetal growth restriction [56–59], increased hypothalamopituitary adrenal axis activity [57, 60], hypertension [61], and reduced brain growth with delayed myelination [62–68]. 

One of the mechanisms known to regulate fetal exposure to active glucocorticoid is through the activity of the 11 beta-hydroxysteroid dehydrogenase (11*β*-HSD) enzyme family, which consists of two known isoforms, 11*β*-HSD-1 and -2. Transfer of glucocorticoids from the maternal to the fetal circulation is regulated by the level of activity of 11*β*-HSD enzyme isoforms in the placenta [69]. Moreover, local tissue availability of glucocorticoid in the fetus is regulated by tissue-specific expression of these enzymes throughout gestation [70]. 11*β*-HSD-1 primarily converts biologically inert cortisone to cortisol (active form) [71, 72]. 11*β*-HSD-1 has both dehydrogenase and reductase activity and is predominantly expressed in tissues with abundant expression of GR such as brain and liver [73]. Conversely, 11*β*-HSD-2 has only dehydrogenase activity and is responsible for converting cortisol to cortisone, thereby preventing inappropriate activation of mineralocorticoid receptors by cortisol and allowing selective access of aldosterone, particularly in the kidney and colon [74]. Although both isoforms of 11*β*-HSD are expressed in the placenta, 11*β*-HSD-2 is the predominant form and its gene expression doubles from approximately 29 to 38 weeks of gestation in humans [75]. 11*β*-HSD-2 appears to be the predominant form in the placenta of humans and guinea pigs [76, 77] and, thus, plays an important role in protecting the fetus from exposure to excess cortisol from the maternal circulation [78]. In sheep, the 11*β*-HSD-1 mRNA transcript predominates over that of 11*β*-HSD-2 [79]; however, both isoforms appear to be equally active in converting cortisol to cortisone in this tissue [80], as in the sheep placenta, 11*β*-HSD-1 has mostly dehydrogenase activity [80]. In the ovine placenta, 11*β*-HSD2 activity decreases between 128 and 132 days and term (*~*145 days of gestation) correlating with the normal prepartum increase in fetal plasma cortisol. Moreover, exogenous administration of cortisol before the endogenous surge reduces 11*β*-HSD2 activity [79]. Hence, fetal cortisol regulates placental 11*β*-HSD2 activity in fetal sheep during late gestation, which has important implications for fetal development before term [79]. Increases in fetal plasma cortisol concentrations induced by adverse intrauterine conditions before term may reduce placental 11*β*-HSD2 activity, thereby enhancing placental exposure to both fetal and maternal cortisol and increasing access of maternal cortisol to fetal tissues [79]. For example, inhibition of the 11*β*-HSD-2 enzyme within the rat placenta increases maternal cortisol transfer to the fetus, and this has been shown to lead to hyperglycaemia and cardiovascular abnormalities in adult rat offspring [81]. 

Exposure of the fetus to glucocorticoids is also regulated by specific transporters that actively transport glucocorticoids. These are located in the placenta and other organs such as the liver and brain. Their expression can determine the degree and duration of fetal or organ specific exposure to glucocorticoids. P-glycoprotein (P-gp, gene symbol *ABCB1/MDR1*) and breast cancer resistance protein (BCRP, gene symbol *ABCG2/BCRP1*) are members of the ATP binding cassette (ABC) membrane transporters [82]. P-gp and BCRP are expressed in multiple organs such as placenta, brain, liver, and intestine [83, 84], where they are responsible for facilitating excretion of a broad range of drugs. MDR1 is present in the human placenta, and its expression decreases with advancing gestation in humans [85] and guinea pigs [86]. Studies in mice have shown that knocking out either P-gp or BCRP results in higher concentrations of their substrates in the fetus because they are efflux transporters located on placental trophoblast cells facing the maternal intervillous space [87, 88]. The substrate specificity of P-gp is similar, but distinct from that of BCRP [82]. For example, P-gp transports substrates such as endogenous glucocorticoids (cortisol and aldosterone) [89] and synthetic antenatal glucocorticoids (betamethasone and dexamethasone) [90], but it is not clear if these are substrates for BCRP [91]. Glyburide, an alternative to insulin for treatment of gestational diabetes, is a substrate for BCRP, while selective serotonin reuptake inhibitors are substrates for P-gp [91]. Due to the different drug transport profile of P-gp and BCRP, regulation of their expression in response to IUGR may be different. To understand the degree and duration of exposure to glucocorticoids in the IUGR fetus, it is necessary to understand the impact of IUGR on the enzymes that control cortisol availability and the expression of drug transporters that remove glucocorticoids from the fetal compartment.

## 3. Is the IUGR Fetus at Risk of Greater Exposure to Antenatal Glucocorticoids?

### 3.1. Alterations in the Placenta of the IUGR Fetus

11*β*-HSD-2 plays an important role in protecting the fetus from exposure to maternal endogenous glucocorticoids, but as stated above, synthetic antenatal glucocorticoids are not substrates for this enzyme. Therefore, maternally administered betamethasone and dexamethasone are able to cross the placenta and act on fetal organs. Interestingly, decreased placental 11*β*-HSD-2 has been reported in response to maternal undernutrition in rats or maternal hypoxia in humans [93, 94], two causes of IUGR, suggesting that the IUGR fetus may have increased exposure to endogenous glucocorticoids. In addition, single but not repeated doses of dexamethasone result in lower placental 11*β*-HSD-2 gene expression in IUGR sheep fetuses when compared to controls [59], suggesting that exogenous glucocorticoids may also affect the enzymatic mechanisms that regulate endogenous glucocorticoids and hence fetal exposure to glucocorticoids. 

**Figure 1:**
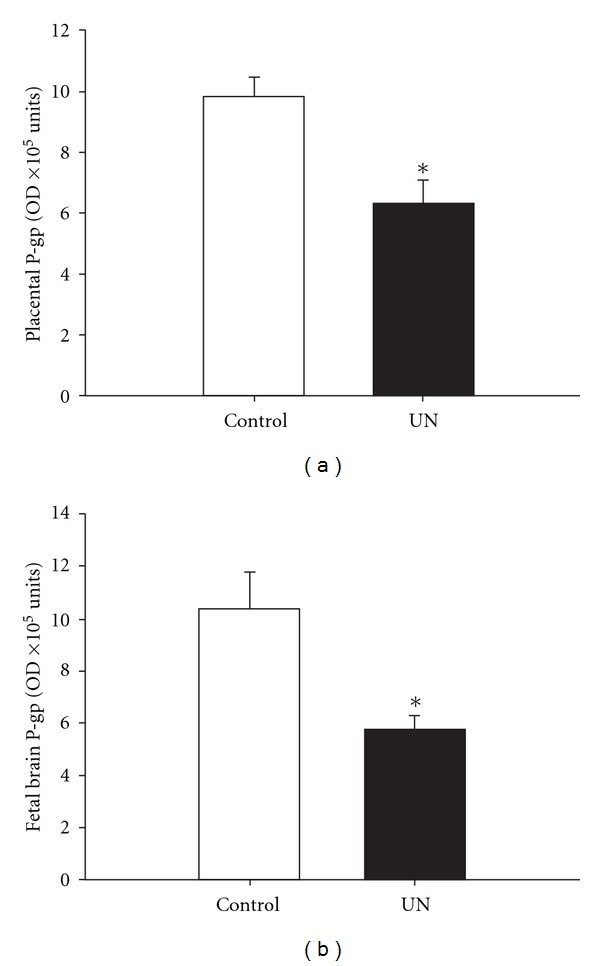
Placental (a) and fetal brain (b) P-gp protein expression in control (open bar, *n* = 6) and maternal undernutrition (UN; filled bar, *n* = 7) at 60–62 days of gestation (term, 69 days) in the guinea pig. P-gp expression (mean ± SEM) was quantified by Western blotting with monoclonal C219 antibody. There was less P-gp protein in the UN placenta and fetal brain than in controls. *P < 0.05. The figure is reproduced with permission from [92].

**Figure 2:**
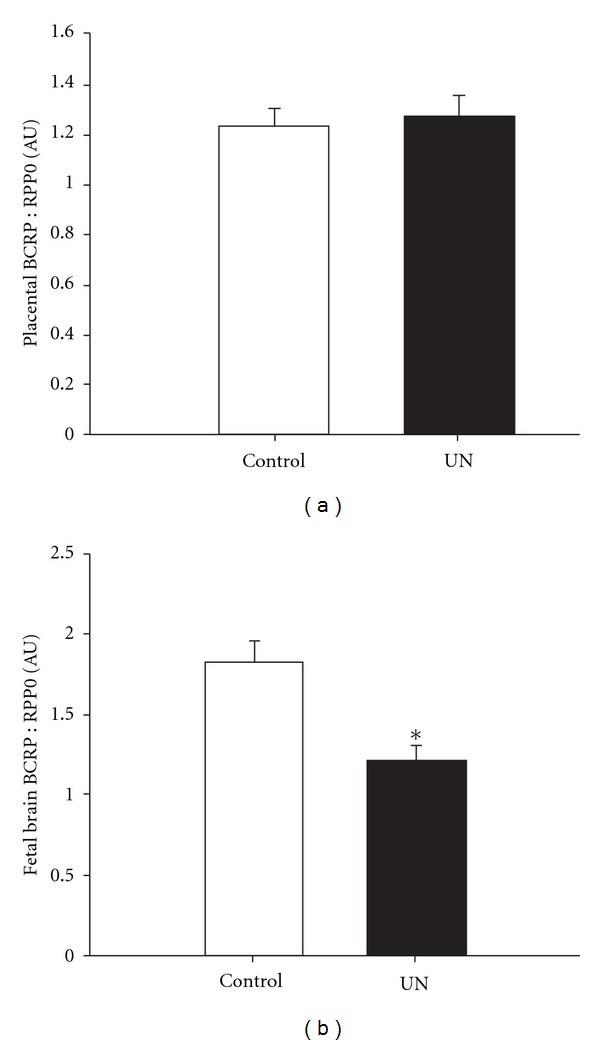
Placental (a) and fetal brains (b) BCRP gene expression in control (open bar, *n* = 6) and maternal undernutrition (UN; filled bar, *n* = 7) at 60–62 days of gestation (term, 69 days) in the guinea pig. BCRP gene expression (mean ± SEM) was quantified by real-time PCR. There was a decrease in BCRP gene expression in the UN fetal brain but not the placenta compared with controls. *P* < 0.05.

Furthermore, work from our laboratory also suggests that IUGR may alter the expression of placental drug transporters. For example, we have demonstrated that maternal undernutrition in guinea pigs significantly reduces placental P-gp protein expression (Figure 1(a)) [92], but does not change placental BCRP gene expression (Figure 2(a)). The reduced expression of P-gp protein in the placenta may lead to increased susceptibility to antenatal glucocorticoid in IUGR fetuses, as a decrease in P-gp protein expression directly correlates with a decreased ability to remove substrates of P-gp from the fetal compartment [95]. Moreover, this situation may be further exacerbated as antenatal glucocorticoids have also been shown to downregulate both P-gp mRNA and protein expression in mice and guinea pigs [86, 96]. Similarly, pregnant mice exposed to dexamethasone have lowered placental BCRP mRNA and protein expression [96]. While it is not clear how the change in P-gp protein expression is regulated in the IUGR fetus, particularly as there is no effect of maternal undernutrition on MDR1 gene expression [92], it is clear that antenatal glucocorticoid treatment of the IUGR fetus is likely to lead to a significant overexposure. Currently, it is unknown whether there are changes in other transporters that regulate transfer of substances between the mother and the IUGR fetus. 

### 3.2. Alterations in the Blood Brain Barrier in the IUGR Fetus

Exposure of the normally grown fetus to antenatal glucocorticoids can cause a decrease in brain growth and maturation [65, 68, 97, 98], but not in nutrient transport [99, 100] or protein synthesis [66]. In sheep, both exogenous and endogenous glucocorticoids decrease blood brain barrier permeability in the sheep fetus at 60% but not 90% of gestation [101]. In addition to its role in the placenta, P-gp is an important component in protecting the fetal brain from exposure to drugs [102]. Brain sparing, defined as an increased brain to body weight ratio, is a notable characteristic of IUGR babies; yet little is known about the impact of IUGR on the expression of drug transporters on the blood brain barrier. In contrast to the effects in the placenta, dexamethasone increases P-gp mRNA and protein expression in rat brain endothelial cells *in vitro* [91, 103]. Similarly, BCRP mRNA and protein expression in rat brain endothelial cells increases in response to dexamethasone *in vitro* [103]. However, it is not known whether there will be similar changes in P-gp expression in the brain of IUGR fetuses because they already have elevated plasma cortisol concentrations [44, 104]. If P-gp expression in the blood brain barrier is altered by IUGR, this has implications for the potential toxicity of drugs used in the management of preterm delivery, maternal hypertension, gestational diabetes, and viral infections. For example, we have shown that IUGR as a result of maternal undernutrition before conception and throughout pregnancy in the guinea pig results in decreased P-gp protein [92] and BCRP; mRNA expression in the brain (Figures 1(b) and 2(b)). Hence, administering dexamethasone or betamethasone to the preterm IUGR fetus may further decrease the protective effects of P-gp and BCRP, although, further studies are required to verify this. 

In conclusion, the ability of the placenta or the blood brain barrier to remove glucocorticoids from the fetal compartment or the brain may be compromised in the IUGR fetus. As a result, depending on the cause of IUGR, that is, maternal undernutrition or placental insufficiency, the IUGR fetus may be exposed to higher endogenous glucocorticoids, as a consequence of IUGR or due to less removal from the brain or fetal compartments, and higher concentrations of antenatal glucocorticoids for a longer period of time than the normally grown fetus. We now turn our focus to the impact of this potentially higher glucocorticoid exposure on organ development in the IUGR fetus. 

## 4. Controversy Regarding the Effectiveness of Antenatal Glucocorticoids on Neonatal Cardiorespiratory Outcomes in the IUGR Fetus 

Despite the established benefits of antenatal glucocorticoids for neonatal lung function in normally grown premature infants, there is considerable controversy about their effectiveness in IUGR fetuses as outlined in the introduction. Moreover, antenatal glucocorticoid therapy applied to the normally grown premature infant is also associated with negative cardiovascular outcomes in later life, as evidenced by an increased blood pressure, in adolescents [105]. This is substantiated by animal studies which demonstrate an increased vascular resistance, fetal blood pressure and a concomitant decrease in cerebral blood flow [106–109]. However, the IUGR fetus adapts to a decreased substrate supply by slowing its growth and undergoing important cardiovascular adaptations [5, 110]. These adaptations are in opposition to those that occur in response to antenatal glucocorticoids. Hence, the question arises as to whether antenatal glucocorticoid therapy of IUGR fetuses will firstly provide the same lung maturational benefit as for normally grown fetuses and secondly whether it will compromise their cardiovascular development.

Below we first examine the role of glucocorticoids in lung development, focusing particularly on surfactant production in both the normally grown and IUGR fetus, before we turn our attention to their role in cardiovascular development, focusing on blood pressure regulation and cardiomyocyte development in the normally grown and IUGR fetuses. 

### 4.1. Mechanism of Glucocorticoid-Induced Surfactant Protein Production

**Figure 3:**
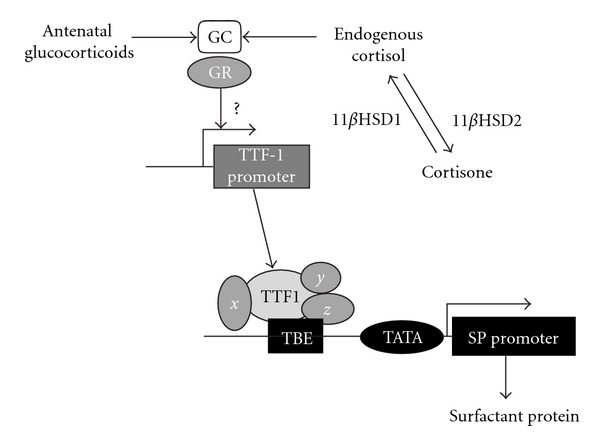
Diagrammatic representation of the mechanism by which endogenous (circulating or locally produced) cortisol and antenatal glucocorticoids act in the lung to increase the gene and protein expression of surfactant protein. GC: glucocorticoid; GR: GC, receptor; TTF-1: thyroid transcription factor-1; TBE: TTF-1 binding element; *x*, *y*, and *z* indicate cofactors for different SP genes.

In both the sheep and human fetuses, plasma cortisol concentrations increase with gestational age [113] and cortisol binds to GR in many target tissues, including the lung. Once the receptor-ligand complex is formed in the cytosol, it translocates to the nucleus where it has the ability to bind to glucocorticoid response elements (GREs) on target genes to alter their expression. Surfactant protein genes contain highly conserved DNA sequences upstream of the transcription site, which are necessary for promot activity in lung epithelial cells *in vitro* [114, 115]. Since the promoters for the surfactant protein genes do not contain an GRE, the stimulatory actions of glucocorticoids on surfactant production are indirect. An alternative indirect response to glucocorticoid signalling may be due to altered transcription factor and/or cofactor activity which is vitally important to surfactant protein regulation [116, 117]. The promoter regions of the 4 surfactant protein genes contain some regulatory elements that are similar among, and others that are different between and hence specific to, the genes. A potential mechanism may be via thyroid transcription factor-1 (TTF-1), a transcription factor that is vital for normal lung development [118]. TTF-1 is primarily expressed by type II alveolar epithelial cells in the fetal lung at term and in postnatal life, and this expression profile is consistent with the developmental pattern of surfactant protein-B expression [119]. It has been proposed that TTF-1 interacts with various cofactors and binds to TTF-1 binding elements (TBEs) expressed on the promoter region of SP-A, -B, and -C gene constructs (Figure 3) [120–122]. In the case of SP-D, TTF-1 regulates gene transcription indirectly via interaction with nuclear factor of activated T cells (NFATs) and other transcription factors [123]. A reduction in TTF-1 abundance has been observed in areas of haemorrhage and atelectasis in infants with RDS and bronchopulmonary dysplasia [119]. Interestingly, in parallel to induction of surfactant protein expression, glucocorticoids have been shown to induce TTF-1 expression in the lung. In a model of fetal rat lung hypoplasia, prenatal dexamethasone treatment significantly increases TTF-1 and SP-B mRNA expression and is associated with increased TTF-1-binding activity to the SP-B proximal promoter [124]. Thus, TTF-1 is an activator of lung-specific genes [125], suggesting an important role for TTF-1 as a transcriptional regulator of indirect glucocorticoid responsiveness in the fetal lung. 

### 4.2. Controversy Regarding the Expression of Surfactant Protein in the Fetal Lung in Sheep Models of IUGR

**Figure 4:**
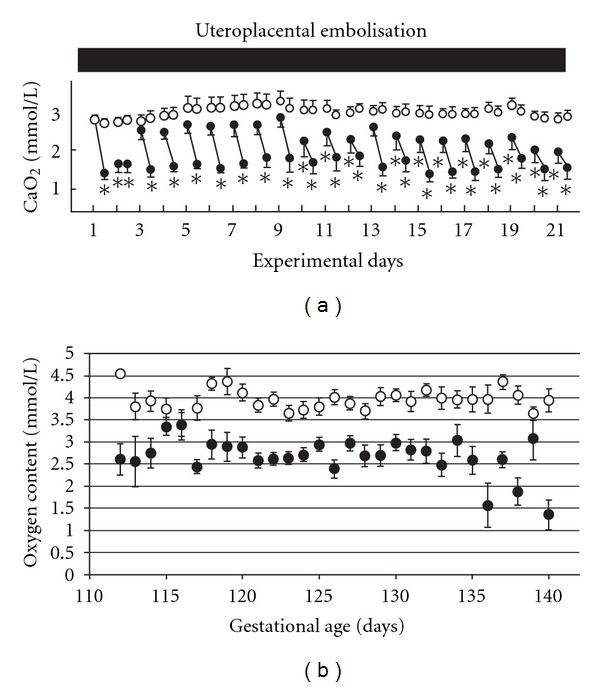
Fetal arterial oxygen content (mmol O_2_/L blood) in two sheep models of human IUGR. The uteroplacental embolisation model of IUGR in the sheep fetus results in periods of fluctuating hypoxemia over the 20-day experimental period starting at 110 days of gestation (a). In contrast, placental restriction (PR, *n* = 28; control, *n* = 31) in sheep (b) results in chronic hypoxemia that is maintained throughout late gestation [5]. Control, open circles UPE (a) or PR (b), closed circles. PR: placental restriction. The figure is reproduced in modified form with permission from [5, 111, 112].

There has been significant controversy about the impact of IUGR on surfactant maturation [33]. Early studies suggested the possibility that the elevated plasma cortisol in IUGR fetuses [44, 104] may represent the mechanism for the stimulation of surfactant maturation. However, different models of IUGR lead to different outcomes in terms of surfactant maturation, some of which correlate with changes in cortisol and others which do not [126]. For example, IUGR induced by uteroplacental embolisation for 21 days during late gestation (~109–130 days; term 150 days) resulted in a decrease in fetal and lung growth and in lung DNA content. However, gene expression of surfactant proteins SP-A and -B in the fetal lung was significantly increased and strongly correlated with fetal plasma cortisol concentrations measured during the last two days of the protocol [127]. In direct contrast, another study on the effect of uteroplacental embolisation for 20 days during a later window in gestation (120–140 days) found that there was no change in gene expression of SP-A, -B, or -C in the fetal lung [128]. In this later study, there was also no correlation between surfactant gene or protein expression and plasma cortisol concentrations. In a third model of IUGR in the sheep fetus, single uterine artery ligation (SUAL) was associated with increased plasma cortisol concentrations, but not with changes in surfactant protein gene expression [129]. Clearly the timing of the insult relative to gestational age, the duration of hypoxemia, and the magnitude and timing of the cortisol response, are all crucial in eliciting the SP or SP mRNA expression response. Given the lack of a relationship between the cortisol and the SP responses, it is possible that the impact of IUGR on lung surfactant protein production may be more dependent on the frequency, degree, and duration of hypoxemia that is induced by different experimental models (Figure 4) rather than the hypercortisolemia induced by the experimental protocol. 

### 4.3. Impact of Chronic Hypoxemia throughout Gestation on Lung Surfactant Protein Production

**Figure 5:**
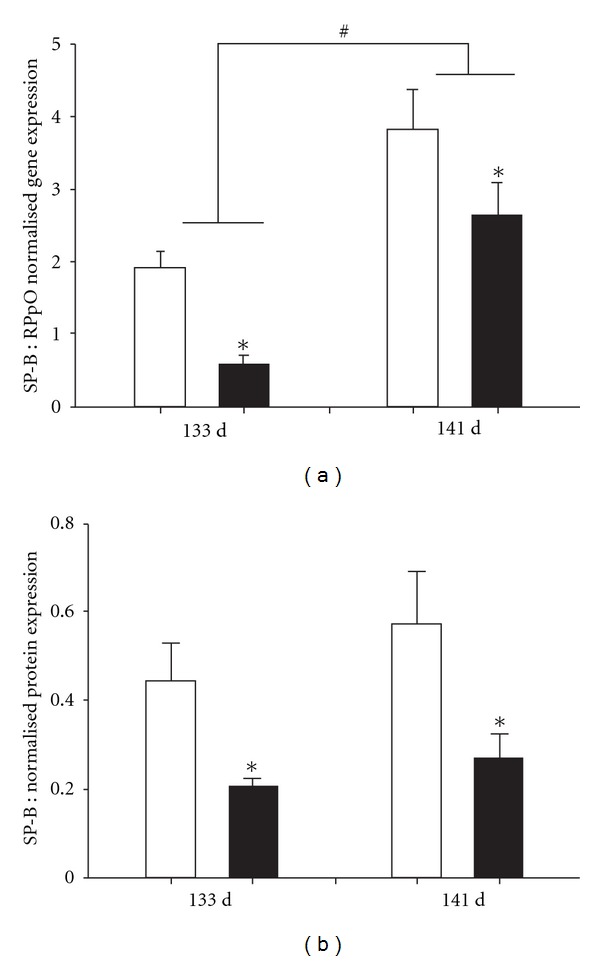
There is a decrease in gene (a) and protein (b) expression of SP-B (as well as for SP-A and -C, data not shown) in the lung of the chronically hypoxemic, PR sheep fetus (black bars) relative to the normally grown sheep fetus (open bars). *P < 0.05 control versus PR; ^#^ *P* < 0.05 gestational age. PR: placental restriction. The figure is reproduced with permission from [126].

Our laboratory has extensive experience using a sheep model of IUGR in which most of the potential placental implantation sites are removed from the uterus of the ewe (carunclectomy) prior to mating, resulting in the subsequent restriction of placental growth and substrate supply, including oxygen and glucose, to the fetus [5, 110]. As a result, the placentally restricted (PR) fetus is chronically hypoxemic, hypoglycaemic, and smaller, although brain growth is spared. This profile directly parallels that observed in the growth-restricted human fetus [110, 130]. Our published work shows that these chronically hypoxemic PR fetuses have lower gene and protein expression of lung SP-A, -B, and -C in late gestation (133 and 140 days of gestation, Figure 5) [126]. Hence, there may be factors present in the lung of the PR fetus which suppress surfactant synthesis.

### 4.4. Role of Endogenous and Potential Risk of Exogenous Glucocorticoids on Surfactant Protein Production in the IUGR Fetus

**Figure 6:**
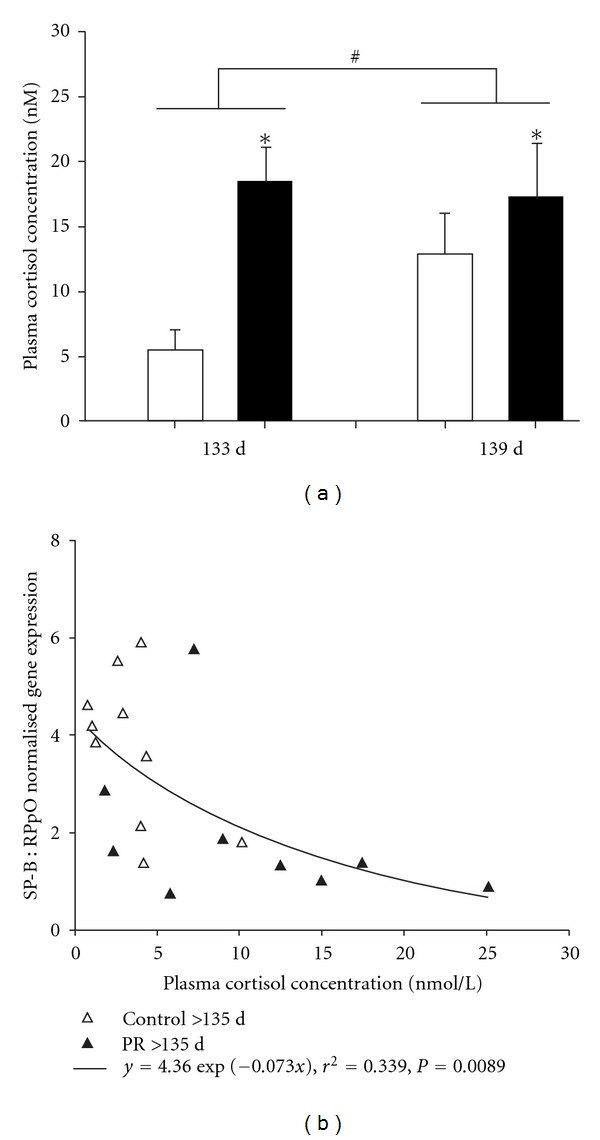
The chronically hypoxemic PR sheep fetus (black bars) has higher plasma cortisol concentrations than the normally grown fetus (open bars) (a). Relative surfactant protein B (SP-B) mRNA expression is inversely correlated with plasma cortisol concentration (b). The figure is reproduced with permission from [126].

Acute increases in plasma cortisol after 21 days of umbilicoplacental embolisation are associated with increased surfactant protein in the lung; however, the timing of this increase may not relate to the timing of preterm birth and thus may not improve neonatal outcomes. Similarly, the PR fetus has higher plasma cortisol concentrations in late gestation compared to the normally grown fetus [104, 111, 129] (Figure 6). However, our data suggest that the increased plasma cortisol concentrations are not sufficient to increase surfactant protein gene or protein expression in the lung of the PR fetus. Hence, further treatment with exogenous glucocorticoid is unlikely to be effective in these fetuses. In contrast, in significantly younger SUAL fetuses (114 days of gestation), betamethasone increases SP-A, -B, and -C mRNA expression despite increased plasma cortisol concentrations [129], but these fetuses may not be chronically hypoxemic. The impact of placental insufficiency on the expression of 11*β*-HSD-1 and -2 or on the GR, and hence the exposure of the lung to glucocorticoid, is currently not known. However, maternal undernutrition increases GR and 11*β*-HSD-1 [132], but umbilical cord occlusion increases 11*β*-HSD-1 and decreases 11*β*-HSD-2 mRNA expression in the lung of sheep fetuses [133]. Further studies are required to elucidate the regulatory mechanisms of surfactant protein gene and protein expression in response to antenatal glucocorticoids in different models of IUGR. 

### 4.5. Impact of Antenatal Glucocorticoids on Cardiovascular Development in the Normally Grown Fetus

Antenatal glucocorticoids can compromise cardiovascular function depending on the type of glucocorticoid and the dose. In sheep, glucocorticoids decrease fetal cerebral blood flow and oxygen delivery due to increased cerebrovascular resistance, and this may explain the decreased brain growth observed at term in human fetuses following either single or repeated glucocorticoid administration [64]. Infusion of glucocorticoids for 48 hours results in peripheral vasoconstriction and an increase in blood pressure of 8–10 mmHg in the late gestation sheep fetus [134, 135]. Furthermore, cortisol infusion to the fetus at 129 day for 5 days increases blood pressure to a level observed in 140-day gestation fetuses as well as increasing plasma concentrations of the vasoconstrictor, angiotensin II [136]. The immediate rise in blood pressure is renin-angiotensin-independent, but thereafter is renin-angiotensin-dependent [137]. Cortisol increases both fetal blood pressure and the reactivity of the fetal vasculature to increasing doses of angiotensin II [138], but dexamethasone does not change vascular reactivity to angiotensin II [134]. Thus exposure of the sheep fetus in late gestation to either excess endogenous or exogenous glucocorticoid changes the vascular reactivity to vasoconstrictors. This leads to increased fetal blood pressure and human newborns exposed to multiple courses of glucocorticoids to have elevated blood pressure in the first week of life [139], which persists into adolescence [105].

### 4.6. Alterations in Regulation of Blood Pressure in the IUGR Fetus and the Potential Risk of Antenatal Glucocorticoids

**Figure 7:**
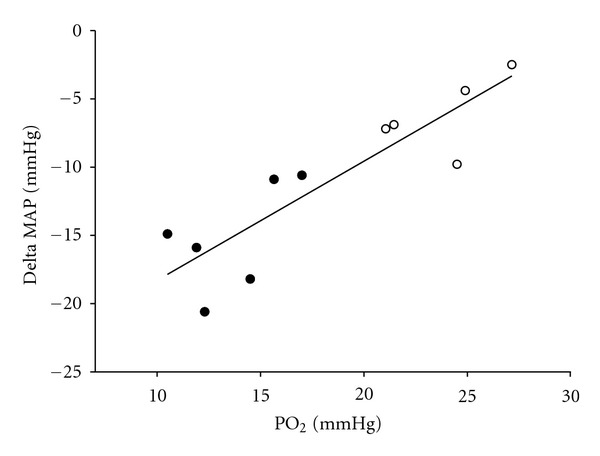
Although blood pressure is the same in the normally grown and the IUGR fetus, the drop in blood pressure in response to an *α* adrenergic antagonist, phentolamine, is related to fetal arterial PO_2_ in control (open circles) and PR (closed circles) fetal sheep (*y* = 0.87 × −27.01, *r* ^2^ = 0.78, *P* = 0.003) [5, 131].

We have reported that although there was no difference in the mean arterial blood pressure between normally grown and IUGR fetal sheep [39], there was a direct relationship between blood pressure and the mean gestational PO_2_ in control animals, which was not present in the IUGR group [140, 141]. Following infusion of phentolamine, an *α*-adrenergic antagonist, in IUGR and control fetuses, we demonstrated that the maintenance of mean arterial pressure in the IUGR fetal sheep depended to a significantly greater extent on the sympathetic nervous system than in control fetuses. This is seen by the direct relationship between the magnitude of the fetal hypotensive response and the fetal arterial PO_2_ (Figure 7). Furthermore, the hypotensive response to *α*-adrenergic blockade was present before the onset of the prepartum cortisol increase [140]. 

Similarly, the maintenance of arterial blood pressure in the IUGR sheep fetus is also more dependent on the renin-angiotensin system than in the normally grown fetus [140, 141]. Infusion of an angiotensin converting enzyme inhibitor after the onset of the prepartum increase in fetal cortisol concentrations from around 135 days of gestation resulted in a greater hypotensive response in IUGR fetal sheep when compared with control fetuses [141]. An earlier activation of glucocorticoid receptors by betamethasone may augment this increase in angiotensin receptors and result in elevated fetal blood pressure that persists into adult life. 

A further adaptation of the IUGR fetus is brain sparing possibly due to the redistribution of blood flow that maintains substrate supply to the brain, adrenals, and heart at the expense of the peripheral organs and tissues [110]. However, it appears that antenatal glucocorticoid exposure alters the fetus' ability to maintain this adaptation to reduced substrate supply. In a recent study in the SUAL sheep model of IUGR, betamethasone caused an equivalent (relative to control) fall in carotid artery blood flow in IUGR fetuses but a large rebound increase in carotid blood flow that was not observed in control fetuses [39]. There was also an increase in cardiac output and blood flow to all organs, particularly the brain in the IUGR fetus [106]. Furthermore, there is also a relationship between cerebral reperfusion and oxidative damage in the brain [39]. These results suggest that the IUGR fetus may be at a greater risk of brain reperfusion injury after treatment with synthetic glucocorticoid than the normally grown fetus [39] due to the cerebral vasodilatory response. 

In summary, therefore, antenatal glucocorticoids may severely compromise the mechanisms established in the IUGR fetus to maintain blood pressure and cerebral blood flow. The ability of the IUGR fetus to survive within a suboptimal environment and to respond appropriately to further impositions is dependent upon the capacity of the fetal cardiovascular system to respond appropriately. The key elements in this response include altered regulation of fetal blood pressure and blood flow to maintain the growth and function of the fetal brain, adrenals, and heart. Any compromise of the fetal cardiovascular system to adapt will clearly have detrimental effects on fetal outcome and challenge fetal survival.

### 4.7. Alterations in Regulation of Cardiomyocyte Development in the IUGR Fetus

Studies of sheep fetuses provide conflicting results regarding the regulation of cardiomyocyte growth by cortisol. In humans, sheep and guinea pigs, the majority of cardiomyocytes present throughout life are present at birth, and, therefore, alterations to cardiomyocytes in late gestation may have a lifelong impact. A comprehensive study of sheep fetuses identified cortisol as a potent cardiomyocyte mitogen [142]. In contrast, a similar intrafetal infusion of cortisol has been reported to decrease DNA content in the left ventricle [143], and adrenalectomized sheep fetuses exhibit greater cardiomyocyte proliferation, thus suggesting that cortisol inhibits progression through the cell cycle [144]. The signalling pathway that links cortisol to proliferation of cardiomyocytes and the question of whether plasma cortisol concentrations which play a role in signalling binucleation [145] remain unclear. 

**Figure 8:**
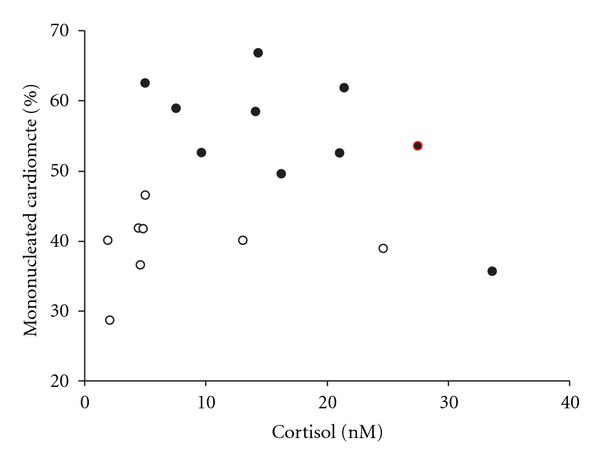
There is no relationship between fetal plasma cortisol concentration and the percentage of mononucleated cardiomyocytes in either control (open circles) or PR (closed circles, carunclectomy model of IUGR in sheep) sheep fetuses.

Studies using two different sheep models of IUGR, both induced by placental insufficiency, have investigated cardiomyocyte development. In both models, placental insufficiency caused a delay in the transition of mononucleated to binucleated cardiomyocytes [146–148] and this is not related to plasma cortisol concentrations in late gestation (140 days, Figure 8). This delay in maturation is in direct conflict with the results from maternal hypoxia studies in rats, which demonstrated an acceleration of binucleation [149]. The difference in findings between the two species reflects the importance of the timing of cardiomyocyte maturation in relation to birth between these species. The use of both sheep models of placental insufficiency highlights how differences in the degree and timing of fetal insults can result in different cardiomyocyte phenotypes. Furthermore, there is an increase in the relative size of cardiomyocytes in the IUGR fetus [148], possibly due to an increase in gene expression of insulin-like growth factor (IGF)-1 receptor and IGF-2 receptor [150], which have hypertrophic effects in cultured cardiomyocytes from the sheep fetus [151]. The added effects of antenatal glucocorticoids on the regulation of cardiomyocyte development in the IUGR fetus remain to be investigated.

## 5. Conclusions

Evidence from our group [92, 126, 140, 141, 148] and others [39, 106, 129, 146, 147] suggests that antenatal glucocorticoids may not have the same effects in IUGR preterm fetuses, as they do in normally grown preterm fetuses. The different responses of the IUGR fetus are likely related to its altered neurohormonal status and the adaptations that the fetus must undergo in the face of reduced substrate supply. Therefore, the effects of antenatal glucocorticoids on the fetus may be dependent on the timing, degree, and duration of hypoxemia, hypercortisolemia, and hypoglycaemia. Furthermore, it is not clear if the benefits of antenatal glucocorticoids outweigh the costs for all fetuses. Of particular concern is the controversy about the effects of antenatal glucocorticoids on lung and cardiovascular function in the IUGR fetus, as the physiological adaptations that this group experiences in response to nutrient and oxygen restriction appear to alter the fetus' ability to regulate endogenous glucocorticoid availability. As a result, these fetuses may be exposed to higher antenatal glucocorticoid concentrations for longer, which may result in an exacerbation of the potentially negative neurological and cardiovascular side effects of antenatal glucocorticoid treatment, possibly without the full capacity to benefit from the lung maturational effects.
